# Postpartum Body Composition in Women With Overweight: Associations With Diet During Pregnancy

**DOI:** 10.1002/osp4.70093

**Published:** 2025-10-13

**Authors:** Ella Muhli, Tero Vahlberg, Lotta Saros, Noora Houttu, Outi Pellonperä, Kristiina Tertti, Kirsi Laitinen

**Affiliations:** ^1^ Integrative Physiology and Pharmacology Unit Institute of Biomedicine University of Turku Turku Finland; ^2^ Department of Obstetrics and Gynecology University of Turku Turku Finland; ^3^ Department of Pediatrics Turku University Hospital Turku Finland; ^4^ Department of Biostatistics University of Turku and Turku University Hospital Turku Finland; ^5^ Department of Obstetrics and Gynecology Turku University Hospital Turku Finland; ^6^ Nutrition and Food Research Center University of Turku Turku Finland

**Keywords:** body composition, diet, obesity, postpartum, pregnancy

## Abstract

**Objective:**

Weight management during the first few years postpartum is considered important for the prevention of future metabolic disturbances. Body fat percentage measured using air displacement plethysmography is an accurate marker of body adiposity. In this secondary analysis of a randomized controlled trial, the aims were to identify distinctive body fat percentage trajectories up to 2 years postpartum in women with overweight and to investigate whether fish oil and/or probiotic supplements, diet, gestational diabetes, or gestational weight gain influenced the body composition.

**Methods:**

Women with overweight or obesity (*n* = 439) were randomized to receive fish oil (1.9 g docosahexaenoic acid and 0.22 g eicosapentaenoic acid) and/or probiotics (*Lacticaseibacillus rhamnosus* HN001 and *Bifidobacterium animalis* ssp. *lactis* 420, 10^10^ CFU each) in a double‐blind manner from early pregnancy until 6 months postpartum. Body composition (*n* = 329) was measured using air displacement plethysmography, at three, six, 12, and 24 months postpartum. Diet in early pregnancy was evaluated using nutrient intakes, dietary patterns, and the Index of Diet Quality (IDQ) questionnaire.

**Results:**

Three postpartum body fat percentage trajectories were identified, but none displayed a sustained decrease in adiposity. A healthy dietary pattern (adjusted OR 5.8 [95% CI 2.5–13.5], *p* < 0.001) and high IDQ score (adjusted OR 2.5 [95% CI 1.1–5.5], *p* = 0.023) in early pregnancy increased the odds of a decreasing trend during the first postpartum year. The fish oil and/or probiotic intervention did not impact the body composition.

**Conclusion:**

Good overall dietary quality during pregnancy may benefit the body composition of women with overweight or obesity at postpartum.

**Trial Registration:**

NCT01922791, ClinicalTrials.gov

**Abbreviations**
FFM: fat‐free mass
FM: fat mass
GDM: gestational diabetes mellitus
GWG: gestational weight gain
IDQ: Index of Diet Quality
IQR: interquartile range
LGMM: linear growth mixture modeling
MET: metabolic equivalent

## 1 Introduction

Obesity is a global health concern; for example in Finland, 20% of childbearing women are living with obesity [1]. It increases the risks of perinatal complications, including the incidence of gestational diabetes mellitus (GDM) [2], which is a well‐established risk factor for type 2 diabetes and cardiovascular diseases [3, 4]. Weight gain between pregnancies has been associated with complications in a subsequent pregnancy [5, 6]. Therefore, weight management during the first few years postpartum is of particular importance, especially for those with prior obesity and/or GDM.

Measuring the body composition provides a more detailed estimate of body adiposity, that is fat mass (FM) and body fat percentage, compared to the typically used measures e.g. weight or BMI. Air displacement plethysmography applies densitometric principles to estimate the body composition; the accuracy of the measurement is considered to be high [7]. Little is known about the evolution of body composition after childbirth. In a study investigating the effects of lifestyle factors on body composition at postpartum [8], it was discovered that during the first 6 months postpartum, the body fat percentage declined more slowly in women with overweight or obesity in comparison to women with normal weight.

The aim of this study was to investigate body fat percentage trajectories at postpartum. This represents a novel and informative approach for studying the development of body composition at this time. The impacts of pregnancy‐associated factors on weight trajectories during the postpartum period have been investigated in a few studies. In a Norwegian cohort, pre‐pregnancy overweight and excess gestational weight gain (GWG) were predictive of a high postpartum weight retention, approximately 5 to 8 kg, at 6 months postpartum followed subsequently by either a marked weight loss or a continued weight gain [9]. In another study [10], women with pre‐pregnancy overweight or obesity had higher odds of either a transient or only a marginal weight loss from one to 12 months postpartum and then a slight weight gain from 12 to 24 months postpartum rather than experiencing a sustained weight loss. In contrast, a higher adherence to a Nordic dietary pattern during pregnancy, including fruits, vegetables, fish, meat, milk, whole‐grain bread, and desserts, has been shown to be associated with a slightly lower postpartum BMI trajectory lasting up to 8 years after the pregnancy [11].

In addition to an overall healthy dietary quality indicated by the dietary pattern, dietary supplementation with probiotics could be beneficial for body composition at postpartum, as indicated by a study where an intervention including dietary counseling and supplementation with probiotics during pregnancy lowered the risk of a greater waist circumference (80 cm or more) at six months postpartum [12]. In the same cohort as studied here, dietary supplementation with fish oil and/or probiotics from early pregnancy onwards did not affect the body composition in late pregnancy [13], but it did reduce the child's risk of being overweight at 2 years of age [14].

This study utilized the following approaches to evaluate postpartum body composition and its pregnancy‐associated determinants in a comprehensive manner: First, distinctive body fat percentage trajectories from three to 24 months postpartum were identified in women with pre‐pregnancy overweight or obesity. Next, the impacts of diet, i.e. dietary quality and dietary patterns, dietary supplementation with fish oil and/or probiotics, GDM, and GWG on the trajectories were investigated. Third, the associations of dietary intake and dietary quality measured in early pregnancy, the fish oil and/or probiotic supplementation, GDM, and GWG on body composition and other anthropometrics at 12 months postpartum were examined. The study hypotheses were that a good dietary quality during pregnancy, as indicated by a high score in the Index of Diet Quality (IDQ) [15] and a healthier dietary pattern [16], would be reflected in a lower postpartum body fat percentage and furthermore that supplementation with probiotics during and after pregnancy would associate with reduced adiposity at postpartum. In addition, it was hypothesized that ideal GWG would associate with a lower adiposity at postpartum, whereas a GDM diagnosis would associate with a higher adiposity at postpartum.

## 2 Materials and Methods

### 2.1 Study Participants

Body composition from three to 24 months postpartum was studied within a single‐center dietary intervention trial investigating the effects of fish oil and/or probiotic supplements during pregnancy on maternal and offspring health [17] (ClinicalTrials.gov: NCT01922791, https://www.clinicaltrials.gov/ct2/show/NCT01922791). The trial was executed at the Turku University Hospital and University of Turku in Finland. Recruitment was organized between October 2013 and July 2017 by distributing information about the study in maternity health clinics, media and social media. The main outcomes of the trial were the incidence of GDM and allergy in the offspring. The inclusion criteria were self‐reported pre‐pregnancy BMI ≥ 25 kg/m^2^ and early pregnancy (< 18 weeks of gestation), and the exclusion criteria were multifetal pregnancy or the presence of metabolic or inflammatory diseases, including type 1 or type 2 diabetes, celiac disease, inflammatory bowel disease, or GDM already diagnosed during the current pregnancy.

**FIGURE 1:**
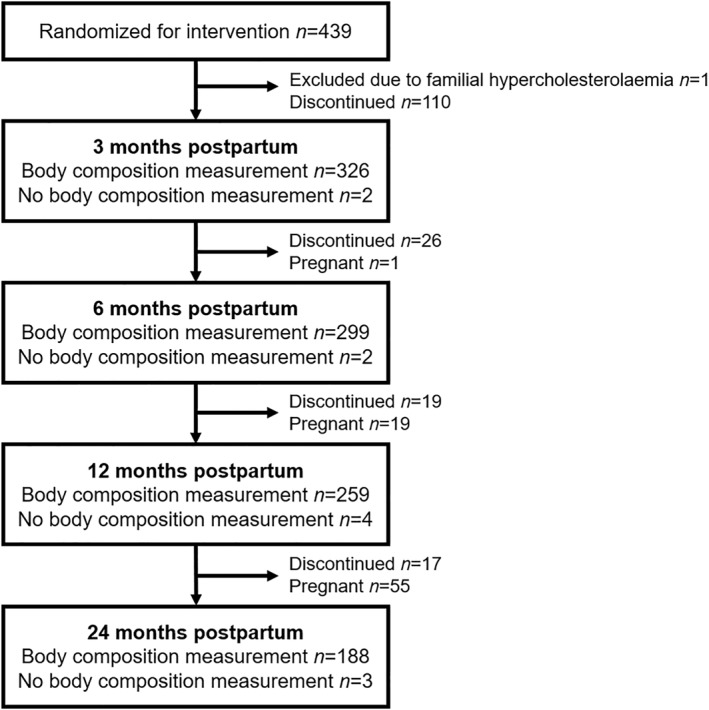
Flow chart of participation in the study visits after the pregnancy.

A total of 439 women were randomized for the intervention with fish oil (1.9 g docosahexaenoic acid and 0.22 g eicosapentaenoic acid), or probiotics (*Lacticaseibacillus rhamnosus* HN001, previously *Lactobacillus rhamnosus* HN001, and *Bifidobacterium animalis* ssp. *lactis* 420, 10^10^ colony‐forming units each), fish oil and probiotics or placebo from the first study visit at approximately 14 weeks of gestation until 6 months postpartum. Of the women, 329 (75%) had at least one body composition measurement (FM, fat‐free mass (FFM), and body fat percentage) after the pregnancy and were included in this report's analyses; these were predefined secondary outcomes of the original trial. Details of the available data for this report are shown in Figure 1. The women participated in two study visits while pregnant, the first in early pregnancy at a median 14.0 weeks of gestation [interquartile range, IQR 12.7, 15.4] and the second in late pregnancy at a median 35.1 weeks of gestation [IQR 34.6, 35.9]), and then in four study visits after the birth of their child at three, six, 12 and 24 months postpartum. In total, 137 women discontinued from participating in the study between the study visits at three and 24 months postpartum due to a new pregnancy or other reasons (Figure 1). The participants completed questionnaires and were interviewed about their background characteristics, including health, education, and smoking habits.

The study was conducted according to the guidelines laid down in the Declaration of Helsinki and all procedures involving the participants were approved by the Ethics Committee of the Hospital District of Southwest Finland (115/180/2012). Written informed consent was obtained from all participants.

### 2.2 Diet and Physical Activity

The participants completed 3‐day food diaries (two weekdays and one weekend day) during the week preceding the study visit in early pregnancy [18]. The mean daily intakes of energy and macronutrients were calculated from the diaries using computerized software AivoDiet (version 2.0.2.3, Aivo, Turku, Finland), which utilizes the Finnish Food Composition Database Fineli. In addition, dietary patterns were modeled with a principal component analysis based on food intakes from the diaries as previously described [16]. Both healthy and less healthy dietary patterns were identified from the data. A healthy dietary pattern was characterized by a higher consumption of vegetables, fruits, rye bread, fish, margarine and oils, cheeses, and eggs, whereas a less healthy dietary pattern was characterized by a higher consumption of multigrain and wheat bread, pastries, sweets, nuts, seeds, crisps and dried fruits, and beverages [16]. Dietary quality in early pregnancy was evaluated using the validated Index of Diet Quality (IDQ) questionnaire [15]. The questionnaire contains 18 questions concerning the amount and frequency of consuming foods, namely whole‐grain products, fat containing foods, dairy, vegetables, fruits and berries, products with added sugar, and meal patterns, during the preceding week. The total score ranges from 0 to 15, with scores < 10 being defined as poor dietary quality and scores ≥ 10 as good dietary quality. Leisure‐time physical activity during the week prior to the early pregnancy study visit was also inquired by a questionnaire, described previously in detail [19]. The metabolic equivalent (MET)‐index was calculated from the intensity, frequency, and duration of physical activity, with the total score ranging from 0 to 120 MET h/week.

### 2.3 Gestational Diabetes Mellitus Diagnosis

The diagnosis of GDM was based on a standard two‐hour 75‐g oral glucose tolerance test for pregnant women conducted according to the Finnish Current Care Guidelines [20]. GDM was diagnosed if at least one blood glucose value was at or above the threshold levels: 0 *h* ≥ 5.3, 1 *h* ≥ 10.0 and 2 *h* ≥ 8.6 mmol/L.

### 2.4 Body Composition and Anthropometric Measurements

During the early pregnancy study visit, height was measured to the nearest 0.1 cm using a wall stadiometer. Pre‐pregnancy BMI was calculated from the height and self‐reported weight obtained from maternity health clinic records. In the study cohort, there was a high correlation (*r* = 0.97) between the self‐reported pre‐pregnancy weight and weight measured at the early pregnancy study visit [21]. Body composition was measured on all study visits using air displacement plethysmography and an electronic scale (the Bod Pod system, software version 5.4.0, COSMED, USA Inc.). Women entered the measurement chamber wearing tight underwear and a cap and were instructed not to exercise or shower before the measurement. In addition, the women were instructed to fast, preferably for two to four hours before the measurement, but also those who did not fast (*n* = 154 at three months postpartum, *n* = 45 at six months postpartum, *n* = 42 at 12 months postpartum, and *n* = 46 at 24 months postpartum) were included in the analyses as the fasting was not associated with the body composition measures (data not shown). The thoracic gas volume was measured to lower the error in the measurement of body composition when possible [22], that is, in a total of 80% of the measurements. The density model formulated by Siri [23] was used to calculate body fat percentage at postpartum. Waist and hip circumferences were measured with a measuring tape on the study visits after the pregnancy, and the waist‐to‐hip ratio was calculated from the measurements.

GWG was evaluated from self‐reported pre‐pregnancy weight to the last weight measurement during pregnancy (either in a maternity health clinic or on the late pregnancy study visit). GWG was classified as ideal, inadequate, or excess based on the recommendations of the Institute of Medicine (IOM) [24].

### 2.5 Statistical Analyses

The statistical analysis was performed with IBM SPSS Statistics 28.0 for Windows (IBM SPSS, Chicago, IL, USA), except for Linear growth mixture modeling (LGMM), which was done with PROC TRAJ in SAS 9.4 (SAS Institute Inc., Cary, NC). Two‐sided statistical tests were used in the analyses, and *p*‐values < 0.05 were considered significant. No adjustments were made for multiple testing. For continuous variables, the normality of distributions was assessed from histograms and evaluated with Shapiro‐Wilk's test. The homogeneity of variances was determined using Levene's test. Normally distributed continuous variables were summarized as means and standard deviations and non‐normally distributed continuous variables as medians and interquartile ranges (IQRs). Categorical data were summarized as frequencies and percentages. No imputations regarding missing data were conducted.

Trajectories of body fat percentage were modeled using LGMM, a mixed modeling method which allows missing values, in SAS. The number of latent growth curves was determined by increasing the number of groups in LGMM and comparing the fit indices of the models (Supporting Information S1: Table S1). The optimal number of trajectory groups was decided based on a posterior group probability (the probability of an individual belonging to a trajectory group; a score of > 0.80 was preferred), the class size (the classes that were identified should not comprise less than 5% of the sample) and clinical interpretability. Clinical characteristics were compared between the trajectory groups and those included and excluded from the analyses using one‐way ANOVA/Independent samples *t*‐test for normally distributed continuous variables, Kruskal‐Wallis test/Mann‐Whitney *U*‐test for non‐normally distributed continuous variables and Chi‐square test for categorical variables.

Multinomial logistic regression was used to analyze the associations of diet, intervention with fish oil and/or probiotics, GDM, and GWG with the body fat percentage trajectory groups. The analyses were adjusted for primiparity and duration of breastfeeding because these characteristics exhibited significant correlations with body composition at 12 months postpartum. The analyses were not adjusted for pre‐pregnancy BMI since it intercorrelated strongly with the other variables. Dietary patterns and IDQ were not included in the same models because of an intercorrelation. The “High and stable” trajectory was used as the reference trajectory in the models.

Spearman correlations between daily macronutrient intakes in early pregnancy, which were non‐normally distributed, and body composition, weight, BMI, waist and hip circumferences, and waist‐to‐hip ratio at 12 months postpartum were evaluated. Body composition and the anthropometric measures at 12 months postpartum were compared between IDQ, dietary pattern, intervention, GDM, and GWG groups with Independent samples *t*‐test or one‐way ANOVA for normally distributed variables and Mann–Whitney *U*‐test or Kruskal–Wallis test for non‐normally distributed variables. Linear models adjusted for the dietary intervention (excluding the comparisons between the intervention groups), primiparity and the duration of breastfeeding were also evaluated. Non‐normally distributed variables, that is FM, weight, BMI, waist circumference, hip circumference, and waist‐to‐hip ratio, were log‐transformed for the linear models. Corrections for multiple comparisons were made with the Bonferroni method.

## 3 Results

### 3.1 Clinical Characteristics

The characteristics of the study participants are shown in Table 1. The median pre‐pregnancy BMI was fractionally below 30 kg/m^2^ among the study population. Nearly half of the women were expecting their first child, and almost a third of the women were diagnosed with GDM in the current pregnancy. As shown in Table 2, the mean body fat percentage, median FM, body weight (kg) and waist circumference (cm) were at their lowest at 12 months postpartum. The women from the study cohort included in the analyses had more often a college or university education compared to those excluded (65% and 44% respectively, *p* = 0.002; data not shown).

**TABLE 1:** Clinical characteristics of the study population allocated to the three body fat percentage trajectory groups.

	All	Decreasing and slowly rising	Inter‐mediate and fairly stable	High and stable	*n*	*p* value
	*n* = 329	*n* = 50 (15%)	*n* = 170 (52%)	*n* = 109 (33%)		
Age in early pregnancy (years; mean, SD)	30.8 (4.5)	30.5 (4.0)	31.0 (4.5)	30.6 (4.7)	329/50/170/109	0.60^a^
Pre‐pregnancy BMI (kg m^−2^; median, IQR)	28.7 (26.5, 31.8)	26.2 (25.1, 26.9)	28.1 (26.5, 30.0)	32.8 (30.3, 35.9)	329/50/170/109	< 0.001^b^
Primiparous (*n*, %)	161 (49)	24 (48)	83 (49)	54 (50)	329/50/170/109	0.99^c^
European ethnicity (*n*, %)	323 (98)	49 (98)	165 (97)	109 (100)	329/50/170/109	0.25^c^
College or university education (*n*, %)	211 (65)	35 (70)	111 (65)	65 (61)	327/50/170/107	0.52^c^
Smoked during pregnancy (*n*, %)	13 (4)	0 (0)	8 (5)	5 (5)	326/49/170/107	0.36^c^
Prior GDM (*n*, %)	27 (8)	5 (10)	12 (7)	10 (9)	329/50/170/109	0.76^c^
GDM in the current pregnancy (*n*, %)	93 (29)	10 (20)	44 (27)	39 (36)	322/50/165/107	0.071^c^
Gestational weight gain (*n*, %)					329/50/170/109	0.39^c^
Inadequate	28 (9)	4 (8)	12 (7)	12 (11)		
Ideal	82 (25)	17 (34)	42 (25)	23 (21)		
Excess	219 (67)	29 (58)	116 (68)	74 (68)		
Good dietary quality in early pregnancy (*n*, %)	160 (49)	33 (66)	78 (46)	49 (45)	328/50/169/109	0.029^c^
Healthy dietary pattern in early pregnancy (*n*, %)	163 (51)	36 (72)	89 (54)	38 (36)	321/50/165/106	< 0.001^c^
MET‐index in early pregnancy (MET h/week; median, IQR)	4.8 (3.0, 12.0)	7.5 (3.0, 12.0)	6.2 (2.0, 12.0)	4.8 (3.0, 12.0)	328/50/170/108	0.24^b^
Breastfeeding duration (months; median, IQR)	11.8 (5.3, 15.0)	12.5 (9.3, 15.4)	11.6 (5.9, 15.0)	10.0 (3.6, 14.5)	275/44/142/89	0.069^b^

Abbreviations: GDM, gestational diabetes; IQR, interquartile range; MET, metabolic equivalent; SD, standard deviation.

^a^ One‐way ANOVA.

^b^ Kruskal‐Wallis test.

^c^ Chi‐square test.

**TABLE 2:** Body composition and anthropometric measures in the study population from three to 24 months postpartum.

	*n*	3 months postpartum	6 months postpartum	12 months postpartum	24 months postpartum
Body fat percentage (%), mean (SD)	326/299/259/188	44.0 (5.5)	43.4 (5.9)	42.8 (6.4)	43.6 (6.4)
FM (kg), median (IQR)	326/299/259/188	37.1 (30.9, 44.1)	35.1 (30.1, 42.8)	34.3 (29.3, 43.6)	36.8 (30.4, 44.3)
FFM (kg), mean (SD)	326/299/259/188	47.3 (5.2)	47.1 (5.2)	47.6 (5.0)	47.7 (5.2)
Body weight (kg), median (IQR)	326/299/259/188	83.4 (76.0, 93.6)	82.1 (74.6, 91.8)	81.3 (74.8, 92.2)	84.6 (75.7, 94.1)
BMI (kg m^−2^), median (IQR)	326/299/260/188	30.1 (27.5, 32.7)	29.7 (26.9, 32.6)	29.7 (26.6, 32.8)	30.4 (27.5, 33.7)
Waist circumference (cm), median (IQR)	324/298/260/187	93.0 (87.0, 99.5)	92.0 (85.5, 98.5)	91.6 (86.5, 99.5)	93.0 (87.9, 101.5)
Hip circumference (cm), median (IQR)	324/298/260/186	111.5 (106.5, 118.1)	110.0 (104.5, 118.0)	111.0 (105.1, 118.4)	111.5 (106.5, 118.6)
Waist‐to‐hip ratio, median (IQR)	324/298/260/186	0.83 (0.80, 0.87)	0.83 (0.79, 0.87)	0.82 (0.79, 0.87)	0.84 (0.80, 0.88)

Abbreviations: FFM, fat‐free mass; FM, fat mass; IQR, interquartile range; SD, standard deviation.

### 3.2 Body Fat Percentage Trajectories From Three to 24 Months Postpartum

**FIGURE 2:**
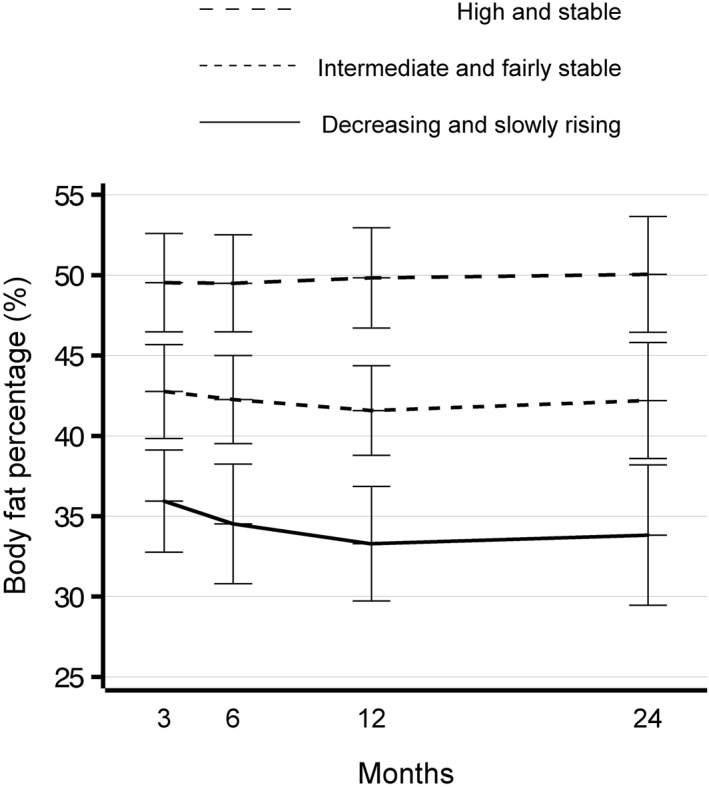
Body fat percentage trajectories from three to 24 months postpartum modeled with Linear growth mixture modeling. “High and stable” (*n* = 109), “Intermediate and fairly stable” (*n* = 170), and “Decreasing and slowly rising” (*n* = 50).

As shown in Supporting Information S1: Table S1, although there could have been many acceptable body fat percentage trajectory group solutions, a three‐trajectory model was ultimately chosen. The trajectories were labeled as follows: “Decreasing and slowly rising” (*n* = 50; quadratic estimate 0.018, *p* = 0.002), “Intermediate and fairly stable” (*n* = 170; quadratic estimate 0.0092, *p* = 0.005) and “High and stable” (*n* = 109; quadratic estimate −0.00079, *p* = 0.84) (Figure 2). On the Decreasing and slowly rising trajectory, body fat percentage decreased from three to 12 months postpartum and then slowly increased from 12 to 24 months postpartum, not quite reaching the same level, approximately 36%, as determined at three months postpartum. On the Intermediate and fairly stable trajectory, the body fat percentage decreased slightly from approximately 43% between three and 12 months postpartum and then increased slightly from 12 to 24 months postpartum. On the High and stable trajectory, the body fat percentage remained stable at nearly 50%. Pre‐pregnancy BMI was at its highest in the High and stable group (Table 1). A healthy dietary pattern and good dietary quality in early pregnancy were most commonly encountered in the Decreasing and slowly rising group.

### 3.3 Determinants of Postpartum Body Fat Percentage Trajectories

Multinomial logistic regression was used to evaluate the associations of dietary quality in early pregnancy, the intervention with fish oil and/or probiotics, GDM, and GWG to the trajectories of body fat percentage. A healthy dietary pattern increased the odds of being on the Decreasing and slowly rising trajectory (adjusted OR 5.8 [95% CI 2.5, 13.5], *p* < 0.001) or on the Intermediate and fairly stable trajectory (adjusted OR 2.0 [95% CI 1.1, 3.6], *p* = 0.024) when compared to the High and stable trajectory. Similarly, a good dietary quality based on the IDQ score increased the odds of being on the Decreasing and slowly rising trajectory compared to the High and stable trajectory (adjusted OR 2.5 [95% CI 1.1, 5.5], *p* = 0.023). Intervention with fish oil and/or probiotics, consumed from early pregnancy onwards, had no statistically significant effects on the trajectories (data not shown).

In the multinomial model including dietary patterns, the women without GDM were more likely to be on the Decreasing and slowly rising trajectory (adjusted OR 2.8 [95% CI 1.1, 7.1], *p* = 0.027) or on the Intermediate and fairly stable trajectory (adjusted OR 2.3 [95% CI 1.2, 4.3], *p* = 0.010) rather than on the High and stable trajectory. Similar results were seen in the model when IDQ classes were utilized instead of dietary patterns; the women without GDM had higher odds of being on the Decreasing and slowly rising trajectory (adjusted OR 2.5 [95% CI 1.0, 6.1], *p* = 0.046) or on the Intermediate and fairly stable trajectory (adjusted OR 2.2 [95% CI 1.2, 4.1], *p* = 0.010) than on the High and stable trajectory. In the model with the dietary patterns included, when compared to the individuals with excess GWG, those with an ideal GWG were more likely to be on the Decreasing and slowly rising trajectory than on the High and stable trajectory (adjusted OR 2.8 [95% CI 1.1, 7.2], *p* = 0.035).

### 3.4 Determinants of Body Composition and Anthropometric Measures at 12 Months Postpartum

The determinants of body fat percentage, FM, FFM, weight, BMI, waist and hip circumferences, and waist‐to‐hip ratio at 12 months postpartum were also investigated. Higher daily protein (*g*) and fiber intake (*g*) in early pregnancy correlated with lower body fat percentage (*r* = −0.15 [95% CI −0.27, −0.02], *p* = 0.017 and *r* = −0.21 [95% CI −0.33, −0.08], *p* < 0.001 respectively). A higher fiber intake in early pregnancy was also associated with lower FM, BMI, and waist circumference (*r* = −0.16 [95% CI −0.28, −0.03], *p* = 0.011; *r* = −0.21 [95% CI −0.33, −0.08], *p* < 0.001; *r* = −0.14 [95% CI −0.26, −0.01], *p* = 0.031, respectively). Protein intake in early pregnancy was associated with a higher FFM (*r* = 0.14 [95% CI 0.02, 0.27], *p* = 0.022).

As shown in Table 3, when compared to the women affected by GDM, those without a GDM diagnosis had a lower body fat percentage and FM at 12 months postpartum. These women also had a lower BMI, weight, waist circumference, and waist‐to‐hip ratio, as well as a lower hip circumference in the model adjusted for the intervention, primiparity, and the duration of breastfeeding. The women with a good dietary quality or a healthy dietary pattern in early pregnancy had a lower body fat percentage, FM and BMI at 12 months postpartum in comparison to the women with a poor dietary quality or a less healthy dietary pattern. In addition, the women with a good dietary quality in early pregnancy had a smaller waist circumference than their counterparts, and the women with a healthy dietary pattern in early pregnancy had a smaller hip circumference than their counterparts. Based on the adjusted model, the women with a healthy dietary pattern also had higher FFM values. GWG or the intervention with fish oil and/or probiotics during and after pregnancy did not affect the body composition or anthropometric measures at 12 months postpartum (Supporting Information S1: Table S2).

**TABLE 3:** Differences in body composition and anthropometric measures at 12 months postpartum between the gestational diabetes groups and the early pregnancy dietary quality and dietary pattern groups.

	GDM	No GDM				Good dietary quality	Poor dietary quality				Healthy dietary pattern	Less healthy dietary pattern			
	*n* = 71	*n* = 166				*n* = 117	*n* = 125				*n* = 116	*n* = 122			
	Estimated marginal mean (95% CI)	Estimated marginal mean (95% CI)	Estimated marginal mean difference (95% CI)	*p* value^a^	*p* value^b^	Estimated marginal mean (95% CI)	Estimated marginal mean (95% CI)	Estimated marginal mean difference (95% CI)	*p* value^a^	*p* value^b^	Estimated marginal mean (95% CI)	Estimated marginal mean (95% CI)	Estimated marginal mean difference (95% CI)	*p* value^a^	*p* value^b^
Body fat percentage (%)	44.4 (42.9, 45.9)	42.0 (41.0, 43.0)	2.4 (0.58, 4.2)	0.009^c^	0.010^d^	41.6 (40.4, 42.8)	43.8 (42.7, 44.9)	2.2 (0.58, 3.8)	0.004^c^	0.008^d^	41.0 (39.8, 42.1)	44.2 (43.0, 45.3)	3.2 (1.5, 4.8)	< 0.001^c^	< 0.001^d^
FM (kg)	38.1 (35.6, 40.8)^e^	33.6 (32.1, 35.1)^e^	1.1 (1.0, 1.2)^f^	0.010^g^	0.002^h^	33.5 (31.8, 35.3)^e^	36.5 (34.6, 38.4)^e^	1.1 (1.0, 1.2)^f^	0.023^g^	0.026^h^	33.0 (31.2, 34.8)^e^	36.7 (34.8, 38.7)^e^	1.1 (1.0, 1.2)^f^	< 0.001^g^	0.006^h^
FFM (kg)	48.3 (47.1, 49.5)	46.9 (46.2, 47.7)	1.3 (−0.074, 2.8)	0.088^c^	0.063^d^	47.6 (46.7, 48.5)	47.3 (46.4, 48.2)	0.28 (−1.0, 1.6)	0.56^c^	0.68^d^	48.1 (47.2, 49.0)	46.8 (45.9, 47.7)	1.3 (0.01, 2.6)	0.15^c^	0.049^d^
Body weight (kg)	86.7 (83.6, 90.0)^e^	81.0 (79.1, 83.0)^e^	1.1 (1.0, 1.1)^f^	0.012^g^	0.003^h^	81.6 (79.3, 84.1)^e^	84.1 (81.8, 86.6)^e^	1.0 (0.99, 1.1)^f^	0.19^g^	0.15^h^	81.6 (79.2, 84.0)^e^	83.8 (81.4, 86.2)^e^	1.0 (0.99, 1.1)^f^	0.071^g^	0.20^h^
BMI (kg m^−2^)	31.2 (30.2, 32.3)^e^	29.1 (28.5, 29.8)^e^	1.1 (1.0, 1.1)^f^	0.001^g^	< 0.001^h^	29.2 (28.5, 30.0)^e^	30.4 (29.7, 31.2)^e^	1.0 (1.0, 1.1)^f^	0.017^g^	0.033^h^	29.2 (28.4, 29.9)^e^	30.3 (29.5, 31.1)^e^	1.0 (1.0, 1.1)^f^	0.002^g^	0.042^h^
Waist circumference (cm)	97.3 (94.9, 99.8)^e^	90.8 (89.3, 92.3)^e^	1.1 (1.0, 1.1)^f^	< 0.001^g^	< 0.001^h^	91.1 (89.3, 93.0)^e^	94.3 (92.4, 96.1)^e^	1.0 (1.0, 1.1)^f^	0.025^g^	0.024^h^	91.6 (89.6, 93.4)^e^	93.4 (91.5, 95.3)^e^	1.0 (0.99, 1.1)^f^	0.097^g^	0.17^h^
Hip circumference (cm)	113.6 (111.3, 116.0)^e^	110.7 (109.2, 112.2)^e^	1.0 (1.0, 1.1)^f^	0.15^g^	0.037^h^	110.5 (108.8, 112.3)^e^	112.8 (111.1, 114.6)^e^	1.0 (1.0, 1.0)^f^	0.10^g^	0.066^h^	110.1 (108.3, 111.9)^e^	113.1 (111.2, 114.8)^e^	1.0 (1.0, 1.1)^f^	0.005^g^	0.025^h^
Waist‐to‐hip ratio	0.86 (0.84, 0.87)^e^	0.82 (0.81, 0.83)^e^	1.0 (1.0, 1.1)^f^	< 0.001^g^	< 0.001^h^	0.82 (0.81, 0.84)^e^	0.83 (0.82, 0.85)^e^	1.0 (0.99, 1.0)^f^	0.45^g^	0.20^h^	0.83 (0.82, 0.84)^e^	0.83 (0.82, 0.84)^e^	1.0 (0.99, 1.0)^f^	0.28^g^	0.53^h^

Abbreviations: CI, confidence interval; FFM, fat‐free mass; FM, fat mass; GDM, gestational diabetes.

^a^ unadjusted.

^b^ adjusted for intervention, primiparity and breastfeeding duration.

^c^ Independent samples *t*‐test.

^d^ Linear model.

^e^ Estimated marginal means from the models presented as geometric means (95% CI).

^f^ Estimated marginal mean differences from the models presented as geometric mean ratios (95% CI).

^g^ Mann‐Whitney *U*‐test.

^h^ Linear model with log‐transformation.

## 4 Discussion

In this study, three distinctive trajectories of the postpartum body fat percentage were identified in women with overweight or obesity, and the influences of several factors, that is, dietary quality, protein and fiber intake, GDM, and GWG, on postpartum adiposity were demonstrated. Intervention with fish oil and/or probiotics, consumed both during and after pregnancy, did not impact the level of adiposity.

To the authors' knowledge, this is the first study examining the trajectories of body fat percentage up to 2 years postpartum. Overall, the majority of the studied women had a fairly high body fat percentage at postpartum, and 15% of the women belonged to the postpartum body fat percentage trajectory, which displayed a decrease from three to 12 months postpartum and subsequently a moderate increase. None of the trajectories showed evidence of a sustained decrease in body fat percentage, but rather remained stable over time. This is notable as it would be beneficial for women with pre‐pregnancy overweight to achieve their pre‐pregnancy or even a lower body fat percentage after pregnancy since this would reduce their future risks for metabolic disorders. In previous studies, pre‐pregnancy overweight, obesity and excess GWG have been associated with a high postpartum weight retention [9, 10, 25].

Interestingly, a healthy dietary pattern in early pregnancy, calculated from the food intake [16], and a good dietary quality, based on a stand‐alone questionnaire [15], increased the odds of being on the Decreasing and slowly rising body fat percentage trajectory at postpartum. Modest correlations between protein and fiber intakes in early pregnancy and body composition at 12 months postpartum were also detected, indicative of the possible beneficial effect of an overall healthy diet and these nutrients on the postpartum body composition. Previously, it was demonstrated [10] that a higher prudent dietary pattern score during pregnancy decreased the risk of a higher postpartum weight retention (approximately 10 kg) at 24 months postpartum. It was stated in another report [11] that a higher adherence to a healthy Nordic dietary pattern during pregnancy was associated with a lower BMI trajectory up to 8 years postpartum. There are similarities in the characteristics of the New Nordic Diet [26] and the healthy dietary pattern characterized by this study group [16], namely a high consumption of fish, vegetables and fruits. The associations between diet in early pregnancy and body composition at postpartum detected here might in part reflect the persistence of healthy dietary habits resulting in long‐term weight management, but also support the proposal that adopting healthy dietary habits in early pregnancy may have long‐term health benefits. Lifestyle habits at postpartum are also of importance. In a recent systematic review [27], it was concluded that intensive dietary and physical activity interventions in the postpartum period might be beneficial for the weight management of women with overweight or obesity.

The effect of combined fish oil and probiotic supplementation during pregnancy on postpartum body composition has not been investigated before. Any impacts of the intervention on body composition or other anthropometric measures at 12 months postpartum were not detected, although adherence to the intervention was reported as being good in this study cohort [17]. In a previous trial [12], dietary counseling and probiotic supplementation during pregnancy lowered the risk of having a waist circumference of 80 cm or more at 6 months postpartum. The cohort investigated there also included women with normal values of pre‐pregnancy BMI. In another study of pregnant women [28], higher plasma levels of long‐chain *n*‐3 fatty acids, a proxy for the intake of fish oil, were associated with lower postpartum weight retention at 18 months postpartum. It is possible that a longer duration of intervention, even initiated prior to pregnancy, here would have had a stronger impact on body composition. Thus, the hypothesized beneficial effect of fish oil and/or probiotic supplements on postpartum anthropometrics remains unresolved.

An ideal GWG according to the IOM recommendations [24] increased the probability of a decreasing trend in body fat percentage during the first postpartum year in this study. However, no differences in body composition or other anthropometric measures were evident at 12 months postpartum between the GWG groups. In addition to postpartum FM retention [29], excess GWG has previously been associated with long‐term abdominal adiposity [30, 31]. In a recent study investigating women with obesity [32], ideal GWG, antenatal moderate or high levels of physical activity, and exclusive breastfeeding for at least 4 months were associated with a negative weight retention from pre‐pregnancy to 6 months postpartum.

A GDM diagnosis increased the odds of being on the High and stable body fat percentage trajectory at postpartum; these women also had a higher body fat percentage, FM and waist circumference at 12 months postpartum when compared to the women without GDM. In another study [33], normal‐weight women with prior GDM had higher abdominal FM and decreased FFM as compared to women without GDM at approximately 4 years postpartum, but these kinds of differences were not seen in women with overweight or obesity. The findings in this study reflect the fact that a high pre‐pregnancy body fat percentage is a significant risk factor for GDM.

The results highlight the need for comprehensive dietary interventions to reduce the gain in FM both during and after pregnancy and to strive to achieve a loss of FM after pregnancy, this being especially valid for women with obesity and GDM. Maternity health clinics may provide a natural setting for the interventions. For example, in Finland, whereas dietary counseling in maternity health clinics is recommended for all pregnant women, it should be particularly targeted for those diagnosed with GDM [20].

The strengths of this study include its longitudinal data collection from a randomized, placebo‐controlled, double‐blind trial, and the precise methodology utilized when measuring adiposity and recording the dietary intake. Multiple confounding factors were recorded and taken into consideration in the statistical analyses. However, the study also has limitations. Most analysis methods were not adjusted for multiple testing. As is typical for longitudinal studies, loss to follow‐up due to discontinuation and new pregnancies might have affected the results, although a mixed modeling method considering missing values was used when determining the body fat percentage trajectories. The women who continued in the study had more commonly a higher educational level. The change in body fat percentage from pre‐pregnancy to postpartum could not be evaluated because the study participants were recruited only during early pregnancy. This should be considered in future studies.

## 5 Conclusion

Three postpartum body fat percentage trajectories were determined in women with overweight or obesity. All the trajectories remained fairly stable during the 21‐month observation period, although a good dietary quality in early pregnancy and ideal GWG increased the likelihood of a decreasing trend in the body fat percentage during the first 12 months postpartum. GDM is associated with a high trend in the body fat percentage. Dietary supplementation with fish oil and/or probiotics from early pregnancy onwards did not influence the body composition at postpartum, but higher protein and fiber intakes had modest beneficial associations. The results indicate a need for comprehensive dietary interventions targeting women with overweight and GDM to help them achieve a lower body fat percentage after or in‐between pregnancies.

## Author Contributions

**Ella Muhli:** data curation, formal analysis, visualization, writing – original draft. **Tero Vahlberg:** formal analysis, visualization, writing – review and editing. **Lotta Saros:** investigation, data curation. **Noora Houttu:** investigation, data curation. **Outi Pellonperä:** investigation, data curation. **Kristiina Tertti:** writing – review and editing, supervision. **Kirsi Laitinen:** conceptualization, methodology, writing – review and editing, funding acquisition, resources, supervision, project administration.

## Conflicts of Interest

The authors declare no conflicts of interest.

## Supporting information


Supporting Information S1
