# Supplementary material for: Postpartum Body Composition in Women With Overweight: Associations With Diet During Pregnancy
Source: Obes Sci Pract. 2025 Oct 13;11(5):e70093. doi: 10.1002/osp4.70093 (PMC12518782; doi:10.1002/osp4.70093)
Supplement: Supplementary file 1 — Supporting Information S1 [file OSP4-11-e70093-s001.docx]

**Postpartum body composition in women with overweight: associations with diet during pregnancy**

Ella Muhli^1,2,3^, Tero Vahlberg^4^, Lotta Saros^1^, Noora Houttu^1^, Outi Pellonperä^2,5^, Kristiina Tertti^2,5^, Kirsi Laitinen^1,6^

^1^Integrative Physiology and Pharmacology Unit, Institute of Biomedicine, University of Turku, Turku, Finland

^2^Department of Obstetrics and Gynecology, University of Turku, Turku, Finland

^3^Department of Pediatrics, Turku University Hospital, Turku, Finland

^4^Department of Biostatistics, University of Turku and Turku University Hospital, Turku, Finland

^5^Department of Obstetrics and Gynecology, Turku University Hospital, Turku, Finland

^6^Nutrition and Food Research Center, University of Turku, Turku, Finland

Contact info: Ella Muhli, elromu@utu.fi, Integrative Physiology and Pharmacology Unit, Institute of Biomedicine, FI-20014 University of Turku, Turku, Finland

**Table S1. Statistics applied in creating the trajectories based on body fat percentage changes from three to 24 months postpartum (n=329).**

|  | Log L. (LL) | AIC | BIC | Group membership (%) |  | Average group posterior probabilities |
| --- | --- | --- | --- | --- | --- | --- |
| 1 group | -3441.19 | -3445.19 | -3452.78 | 100 |  | 1.00 |
| 2 groups | -3185.88 | -3193.88 | -3209.06 | 48.5/51.5 |  | 0.95/0.93 |
| 3 groups | -3046.80 | -3058.80 | -3081.57 | 15.4/50.1/34.5 |  | 0.94/0.92/0.94 |
| 4 groups | -2966.79 | -2982.79 | -3013.16 | 10.2/36.8/37.5/15.5 |  | 0.94/0.92/0.89/0.93 |
| 5 groups | -2921.88 | -2941.88 | -2979.84 | 6.1/16.3/34.0/31.3/12.3 |  | 0.98/0.89/0.87/0.89/0.91 |
| 6 groups | -2893.60 | -2917.60 | -2963.15 | 5.9/12.2/29.5/28.0/19.4/5.0 |  | 0.96/0.88/0.89/0.82/0.86/0.94 |

**Table S2. Body composition and anthropometric measures at 12 months postpartum in gestational weight gain and dietary intervention groups.**

|  | Ideal GWG | Inadequate GWG | Excess  GWG | |  | Fish oil + placebo | Probiotics + placebo | Fish oil + probiotics | Placebo +  placebo | |  |
| --- | --- | --- | --- | --- | --- | --- | --- | --- | --- | --- | --- |
|  | *n*=60 | *n*=23 | *n*=160 |  |  | *n*=66 | *n*=58 | *n*=58 | *n*=62 |  |  |
|  | estimated marginal mean (95% CI) | estimated marginal mean (95% CI) | estimated marginal mean (95% CI) | *p*-value^a^ | *p*-value^b^ | estimated marginal mean (95% CI) | estimated marginal mean (95% CI) | estimated marginal mean (95% CI) | estimated marginal mean (95% CI) | *p*-value^a^ | *p*-value^c^ |
| Body fat percentage (%) | 42.2 (40.6, 43.9) | 44.7 (42.0, 47.4) | 42.6 (41.6, 43.7) | 0.25^d^ | 0.30^e^ | 44.1 (42.5, 45.7) | 41.7 (40.0, 43.3) | 42.4 (40.7, 44.1) | 42.7 (41.1, 44.4) | 0.19^d^ | 0.20^e^ |
| FM (kg) | 34.1 (31.6, 36.7)^f^ | 37.8 (33.3, 42.7)^f^ | 35.0 (33.4, 36.6)^f^ | 0.45^g^ | 0.39^h^ | 37.2 (34.6, 39.9)^f^ | 33.4 (31.0, 36.1)^f^ | 34.3 (31.8, 37.1)^f^ | 35.2 (32.7, 37.8)^f^ | 0.27^g^ | 0.23^h^ |
| FFM (kg) | 47.3 (46.0, 48.6) | 47.3 (45.1, 49.4) | 47.5 (46.7, 48.3) | 0.67^d^ | 0.95^e^ | 47.6 (46.4, 48.9) | 47.4 (46.1, 48.7) | 47.1 (45.8, 48.4) | 47.5 (46.2, 48.8) | 0.88^d^ | 0.95^e^ |
| Body weight (kg) | 82.0 (78.7, 85.4)^f^ | 85.2 (79.7, 91.2)^f^ | 82.9 (80.8, 85.0)^f^ | 0.76^g^ | 0.63^h^ | 85.3 (82.0, 88.7)^f^ | 81.5 (78.1, 84.9)^f^ | 81.9 (78.6, 85.4)^f^ | 83.0 (79.7, 86.4)^f^ | 0.34^g^ | 0.38^h^ |
| BMI (kg m^-2^) | 29.3 (28.2, 30.4)^f^ | 30.9 (29.0, 32.8)^f^ | 29.8 (29.2, 30.5)^f^ | 0.41^g^ | 0.36^h^ | 30.9 (29.9, 32.1)^f^ | 29.2 (28.1, 30.3)^f^ | 29.3 (28.2, 30.4)^f^ | 29.8 (28.8, 30.9)^f^ | 0.13^g^ | 0.10^h^ |
| Waist circumference (cm) | 91.9 (89.3, 94.6)^f^ | 96.4 (92.0, 101.1)^f^ | 92.5 (90.9, 94.1)^f^ | 0.17^g^ | 0.22^h^ | 94.4 (91.9, 97.1)^f^ | 91.7 (89.0, 94.3)^f^ | 92.3 (89.7, 95.0)^f^ | 92.5 (89.9, 95.2)^f^ | 0.49^g^ | 0.47^h^ |
| Hip circumference (cm) | 110.8 (108.4, 113.3)^f^ | 114.4 (110.3, 118.8)^f^ | 111.6 (110.1, 113.2)^f^ | 0.59^g^ | 0.34^h^ | 113.3 (111.0, 115.8)^f^ | 111.5 (109.0, 114.0)^f^ | 110.9 (108.5, 113.5)^f^ | 111.1 (108.6, 113.5)^f^ | 0.69^g^ | 0.48^h^ |
| Waist-to-hip ratio | 0.83 (0.81, 0.84)^f^ | 0.84 (0.82, 0.87)^f^ | 0.83 (0.82, 0.84)^f^ | 0.43^g^ | 0.60^h^ | 0.83 (0.82, 0.85)^f^ | 0.82 (0.81, 0.84)^f^ | 0.83 (0.82, 0.85)^f^ | 0.83 (0.82, 0.85)^f^ | 0.81^g^ | 0.68^h^ |
| CI, confidence interval; FFM, fat-free mass; FM, fat mass; GWG, gestational weight gain  ^a^unadjusted  ^b^adjusted for intervention, primiparity and breastfeeding duration  ^c^adjusted for primiparity and breastfeeding duration  ^d^One-way ANOVA  ^e^Linear model  ^f^Estimated marginal means from the models presented as geometric means (95% CI)  ^g^Kruskal-Wallis test  ^h^Linear model with log-transformation | | | | | | | | | | | |
